# A Role for the Bone Marrow Microenvironment in Drug Resistance of Acute Myeloid Leukemia

**DOI:** 10.3390/cells10112833

**Published:** 2021-10-21

**Authors:** Seyed Mohammadreza Bolandi, Mahdi Pakjoo, Peyman Beigi, Mohammad Kiani, Ali Allahgholipour, Negar Goudarzi, Jamshid S. Khorashad, Anna M. Eiring

**Affiliations:** 1 Department of Immunology, Razi Vaccine and Sera Research Institute, Karaj, Iran; Mreza.bolandi@gmail.com (S.M.B.); Negargoudarzi1374@gmail.com (N.G.); 2 Department of Pharmacology, Karaj Branch, Islamic Azad University, Karaj, Iran; DVM.Mohammadkiani@gmail.com (M.K.); Aliallahgholipour@gmail.com (A.A.); 3 Department of Hematology, Faculty of Medical Sciences, Tarbiat Modares University, Tehran, Iran; pakjoomahdi@gmail.com (M.P.); Peymanbeigi1373@yahoo.com (P.B.); 4 Centre for Haematology, Hammersmith Hospital, Imperial College London, London W12 0HS, UK; j.sorouri-khorashad@imperial.ac.uk; 5 Center of Emphasis in Cancer, Department of Molecular and Translational Medicine, Texas Tech University Health Sciences Center at El Paso, El Paso, TX 79905, USA

**Keywords:** drug resistance, acute myeloid leukemia, bone marrow microenvironment, leukemic stem cell

## Abstract

Acute myeloid leukemia (AML) is a heterogeneous disease with a poor prognosis and remarkable resistance to chemotherapeutic agents. Understanding resistance mechanisms against currently available drugs helps to recognize the therapeutic obstacles. Various mechanisms of resistance to chemotherapy or targeted inhibitors have been described for AML cells, including a role for the bone marrow niche in both the initiation and persistence of the disease, and in drug resistance of the leukemic stem cell (LSC) population. The BM niche supports LSC survival through direct and indirect interactions among the stromal cells, hematopoietic stem/progenitor cells, and leukemic cells. Additionally, the BM niche mediates changes in metabolic and signal pathway activation due to the acquisition of new mutations or selection and expansion of a minor clone. This review briefly discusses the role of the BM microenvironment and metabolic pathways in resistance to therapy, as discovered through AML clinical studies or cell line and animal models.

## 1. Introduction

Hematopoietic stem cells (HSCs) produce all blood cell types throughout life due to their capacity for self-renewal and differentiation [1,2]. Any disruption of this process can lead to abnormal expansion of cellular clones, which may lead to hematologic malignancies such as acute myeloid leukemia (AML) [2,3,4,5]. AML is a heterogeneous disease with extreme proliferation of myeloblasts (>20%) in the bone marrow (BM) [6,7]. AML is responsible for 1% of all annual new cancer cases and 1.8% of all cancer deaths in the United States (US). AML is a male predominant disease, with a risk ratio of 1.6 for males and 1.2 for females [3]. It is among the top 15 most prevalent cancers, with an average age of 70 years at diagnosis [8]. Morbidity and mortality of AML increase with age [9], and the global AML incidence has progressively increased during the last several decades (from 63,840 cases in 1990 to 119,570 cases in 2017) [10]. In children, AML is the most common leukemia after acute lymphoblastic leukemia (ALL), with a five-year survival rate of 64% [11]. The best prognosis among the AML subtypes is acute promyelocytic leukemia (APL), which harbors the t(15;17) translocation, generating the promyelocytic leukemia (*PML*)-retinoic acid receptor alpha (*RARA*) fusion gene, and is curable with arsenic trioxide and all-trans retinoic acid (ATRA) treatment. The worst survival rate among the AML subtypes is in patients with FMS-like tyrosine kinase 3 (FLT3) mutations, monosomy 7, and del 5q [11,12,13]. Moreover, childhood AML prevalence is highest among newborns less than one year of age, with an incidence rate of 18.4 per million [11].

Although a diverse range of treatment options for AML have been introduced over the past several decades, the health care community is still struggling to improve the poor prognosis, especially in elderly patients [14]. The well-known 7+3 induction chemotherapy is the most common approach for non-APL disease, which is based on three days of Anthracyclines (in most cases Daunorubicin) accompanied with seven days of continuous infusion with a pyrimidine analog like Cytarabine [15]. After the achievement of complete remission (CR), hematopoietic stem cell transplantation (HSCT) and/or intermediate to high dose Cytarabine is prescribed as consolidation therapy [16]. However, AML has the shortest overall survival (OS) among the acute leukemias, with a 2-year and 5-year OS of only 32% and 24%, respectively [14]. To be more specific, relapse and primary (initial) refractory AML are indispensable challenges in the treatment of AML. Indeed, 10–40% of younger patients (<45 years) and more than 60% of elderly AML patients (>60 years) are primarily refractory to initial induction chemotherapy. A significant proportion of AML patients relapse, even those who achieve CR. AML relapse is due to various factors, such as dysregulation of the signaling pathways associated with DNA damage response sensing proteins, mutations in cell cycle control genes, changes in programmed cell death (including apoptosis and autophagy), altered anti-cancer drug trafficking, and other mechanisms that still need to be discovered [17,18]. Another important reason why many patients relapse is the inability of most therapies to target the leukemic stem cell (LSC) population [19].

The etiology of AML is not completely understood. AML is generally categorized into three groups: (1) de novo AML (initially diagnosed with AML), (2) secondary AML (myeloid disorders that develop after other diseases, such as myelofibrosis, chronic myeloid leukemia, or myelodysplastic syndromes), and (3) therapy-related AML (t-AML) (following chemical exposure) [20]. AML has been associated with risk factors such as old age, male gender, smoking, chemicals (e.g., benzene and formaldehyde), genetic disorders (e.g., Fanconi anemia (FA) and Bloom syndrome), radiation, AML familial history (mutations in GATA Binding Protein 1 (*GATA1*), DEAD-box helicase 41 (*DDX41*), runt-related transcription factor1 (*RUNX1*), CCAAT/enhancer-binding protein alpha (*CEBPA*), and Ankyrin repeat domain 26 (*ANKRD26*)), as well as chemotherapeutic agents (alkylating agents and topoisomerase II inhibitors) [21]. In the present review, we discuss the various mechanisms contributing to drug resistance in AML, including both intrinsic and extrinsic mechanisms that have been discovered through animal models or clinical investigations.

## 2. Genomic and Immunophenotypic Characteristics

General symptoms of AML include fatigue, shortness of breath, bruising and recurrent infections that are consequences of anemia, thrombocytopenia, and neutropenia [22]. For initial diagnosis, BM aspiration is performed to assess morphology, molecular genetic tests, cytogenetic analysis, cytochemistry (including myeloperoxidase (MPO) activity), and immunophenotyping (e.g., CD34, CD13, CD33, CD113, and CD117) [22]. Metastasis is rarely seen in AML; however, it is mostly related with monocytic lineage infiltration in monoblastic/monocytic AML (AML-M4/M5 FAB category), which may lead to gingival hyperplasia or myeloid sarcoma within the central nervous system (CNS), abdomen, ovaries, muscles, and lungs in AML, especially for patients with the t(8;21) translocation (AML-M2 FAB category) [23,24,25]. 

Genomic analyses have revolutionized AML diagnosis and prognosis [26]. According to the latest world health organization (WHO) categorization, 85% of AML patients show one or more of the genomic abnormalities presented in Table 1 [27]. During the immunophenotypic analysis of AML, CD34 and CD117 are the antigens commonly used to detect myeloblasts [28]. CD13, CD15, CD33, MPO, and CD16 are myeloid markers commonly used for lineage assignment, along with monocytic differentiation markers such as CD11b, CD64, CD14, and CD4 [28]. Erythroid precursors express CD71, CD105, CD117, CD235a, and CD36, whereas megakaryocytic precursors express CD61 and CD42b [28]. In AML, an increase of the immature myeloid population must be confirmed through diagnosis of at least two markers, including MPO, CD33, CDw65, and CD117 [22]. At least one pan myeloid marker (CD13, CD33, and CDw65) is seen in 95% of cases, whereas all three markers can be found in ~50% of cases. Lymphoid markers such as CD3, CD2, CD4, CD5, CD56, CD22, and CD79a are expressed in almost 25% of cases, whereas the CD7 and CD19 markers can be found in 10–30% and <3% of patients, respectively [22,28].

## 3. Treatment

According to European Leukemia Net (ELN), AML prognosis using cytogenetic and molecular analysis is divided into four groups, including favorable, intermediate I, intermediate II, and adverse [29]. From this group, patients older than 60 years of age show the worst prognosis [20]. AML treatment is generally associated with poor outcomes, even in young patients using high dose chemotherapy and HSCT [30]. Drug resistance and low five-year survival is a main feature of AML. In patients <70 years of age, the five-year survival is nearly 40%, but in patients older than 70 years, the three-year survival does not go beyond 10% [3,30,31,32,33]. Recent advances in chemotherapy, immunotherapy, HSCT, and targeted therapy have led to improvements in AML treatment [34]. The 7+3 regimen is the first choice of AML therapy, which includes seven days of Daunorubicin or Idarubicin and 3 days of Cytarabine administration [34,35,36]. This regimen is the most effective approach for patients in the favorable prognosis category (below 60 years and/or with Core binding factor (*CBF*)/Nucleophosmin 1 (*NPM1*) translocation) [20]. Despite its widespread use, this regimen is unfortunately associated with increased toxicity and often fails to eradicate the LSC population, resulting in many cases of relapsed or refractory AML [31,37]. In addition to conventional therapies for AML, novel agents have been introduced due to the identification of underlying genomic abnormalities, such as Midostaurin in the case of AML patients with *FLT3* mutations [20].

HSCT, targeted therapy, or other types of chemotherapy are mainly post-induction treatment strategies based on the patient’s status, AML type, and appropriate HSC donor availability [20]. To perform HSCT, morphologic complete remission (M-CR) must be achieved. M-CR means that blasts in the BM must be less than 5% among at least 200 nucleated cells, there should be no sign of extramedullary or persistent disease, and platelet and neutrophil absolute count must be more than 100,000 and 1000 per microliter, respectively [20]. To monitor minimal residual disease (MRD) and treatment response, methods such as morphologic assessment, multiparameter flow cytometry, digital droplet PCR (ddPCR), real-time quantitative (RTq)–PCR, and next generation sequencing (NGS) are applied [20,38]. For HSCT, standard myeloablative conditioning (MAC-HSCT) regimens in AML include Cyclophosphamide and total body irradiation (TBI) or Cyclophosphamide and Busulfan or Fludarabine and Busulfan [39], which is not recommended in patients older than 70 years due to the possibility of toxicity. Therefore, only a small proportion of patients can benefit from this approach [39,40]. While HSCT is the only definitive cure for AML, it is accompanied by graft-versus-host disease (GVHD) as the most major chronic side effect and the prognosis after HSCT remains poor [40,41,42]. 27–35% of younger patients with de novo AML and 38–62% of patients older than 60 years of age are deprived of HSCT because they fail to achieve M-CR [20].

Poor response to conventional therapies, and the side effects associated with them, have led to diverse therapeutic strategies and novel agents which are hoped to improve survival. Targeted therapy in AML is considered the next game changer of the field when cytogenetic and molecular abnormalities provide an actionable target. The selection of treatment for many cases would be based on the individual characteristics of the disease, indicating personalized medicine as the evolving approach for management of AML cases [43]. Based on this, new inhibitors have been developed according to the known target, such as immunotherapy to target specific intra- or extra-cellular antigens. 

Genomic alterations in *FLT3*, *NPM1*, DNA methyl transferase 3A (*DNMT3A*), tumor protein 53 (*TP53*), TET methyl cytosine dioxygenase 2 (*TET2*), and isocitrate dehydrogenase (*IDH1/2*) are frequently observed in AML [44,45]. In recent years, some new medications, including Midostaurin (FLT3 inhibitor), Gilteritinib (FLT3 inhibitor), CPX-351, Gemtuzumab-Ozogamicin (anti-CD33 monoclonal antibody conjugated with calicheamicin), Enasidenib (IDH2 inhibitor), Ivosidenib (IDH1 inhibitor), Venetoclax (B-cell lymphoma 2 (BCL-2) inhibitor), and Glasdegib (Smoothened (SMO) inhibitor), have been approved by the Food and Drug Administration (FDA) to be used for AML treatment [46], all of which are targeted therapies aimed at personalizing the approach to management of AML [8]. In this approach, drugs are administered based on the patient’s individual condition after molecular analysis, age, clinical status, chemotherapy history, and bone marrow dysplastic alterations are identified [8]. Some promising drugs that inhibit specific markers to overcome AML are shown in Table 2. 

## 4. Resistance

Many patients who achieve CR will relapse in less than three years while exhibiting drug resistance and poor prognosis [49]. Relapse is usually diagnosed via clonal expansion of minor pre-existing clones, or through detection of novel mutations acquired by the leukemic cells, which can be more aggressive if they develop in less than six months following treatment [20]. Drug resistance is usually categorized as primary or secondary (acquired) [34]. Primary drug resistance is usually defined as de novo lack of response to treatment and is related to the patient’s leukaemia genotype, availability of the target for the applied drug, or the G0 cell cycle phase of the LSC population. Secondary resistance, on the other hand, indicates a gradual loss of sensitivity to the drug after an initial response. This is associated with disease evolution through the development of escape mechanisms, such as new mutations which lead to recruiting or blocking signaling pathways, or enhanced production of cytokines, interleukins, or growth factors [34,50].

LSCs remain a major obstacle in the way of achieving complete remission in AML [51,52]. Recent studies have revealed that the leukemic niche plays a crucial role in AML persistence by nesting of LSCs and protecting them from both the immune system and therapeutics [53]. LSCs are considered to be responsible for AML initiation, chemotherapy resistance, disease progression, and MRD due to their quiescence and higher self-renewal capabilities [53,54]. LSCs may originate from HSCs or HPCs that acquire the ability of self-renewal upon oncogenic alterations [55]. Generally, abnormal proliferation, disruption of differentiation, and maturation arrest are consequences of events like *TET2*, *NPM1*, *DNMT3A*, *IDH1*, and *IDH2* mutations, which can turn normal HSCs into pre-leukemic cells and finally leukemic cells [5,56,57]. LSCs may reside at the level of the CD34^+^38^−^ or CD34^+^38^+^ cell fraction [55]. The common specificities of stem cells, such as self-renewal capacity, multi-drug resistance, and immaturity, enable them to initiate leukemia in immunosuppressed mouse models of the disease [58,59]. Specific markers of LSCs have not been completely defined due to the similarities with normal HSCs; however, a variety of expressed markers have been identified among AML patients [59,60]. During leukemic transformation, LSCs deploy various molecules and immune suppressor cytokines to alter vital regulatory mechanisms within the BM microenvironment [61], leading to failure of the immune system to maintain normal hematopoiesis [61]. LSCs escape the effects of cytotoxic agents by nesting in hematopoietic niches within the BM microenvironment [53,62].

AML cells can have a negative influence on normal haematopoiesis. In the beginning, initial leukemic stem cells (pre-LSCs) and HSCs are both located in the same microenvironment. However, leukemic cells gradually occupy and change the hematopoietic niche [63]. Kumar et al. indicated that leukemic cells can mediate molecular changes in the BM niche and convert the normal hematopoietic niche into the leukemic niche, which supports leukemic cell survival and growth [64]. In addition, leukemic cells decrease the capacity of the niche to maintain HSCs and block normal hematopoiesis [13,65]. Xenograft models of AML have shown that CXCR4-expressing leukemic cells compete with normal HSCs to bind CXCL12-expressing BM endothelial cells. This causes a reduction in normal hematopoiesis and a decreased response to therapy, indicating an important role for the BM microenvironment in AML therapeutic responses [66,67]. In AML patients, the expression of the Jagged-1, Hes-1, Hes-5, and NOTCH signaling pathways in mesenchymal stem cells (MSCs) was demonstrated to be reduced, and their co-culture with normal HSCs inhibited normal hematopoiesis [68]. Additionally, alterations of transcription factors (TFs) may be responsible for drug resistance in AML LSCs by upregulating ABC transporters, cell cycle progression molecules, and oxidant protection [53,69,70]. Transcription factors that play an important role in AML drug resistance are listed in Table 3.

## 5. The Normal BM Microenvironment

**Figure 1:**
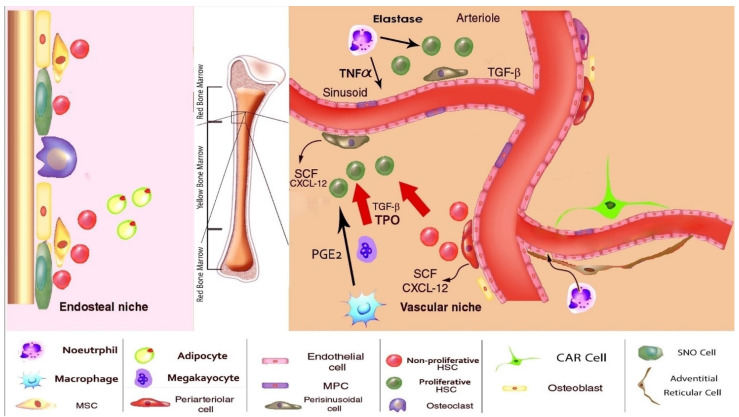
The endosteal and vascular bone marrow niche. The endosteal niche hosts quiescent or self-renewing HSCs. The vascular niche hosts differentiating HSCs using cell-cell interactions and secreted molecules. This figure is adopted from [98]. CAR cells, CXCL12-abundant reticular cells; HSC, Hematopoietic stem cells; MSC, mesenchymal stem cells; MPC, Myeloid progenitor cells; PGE_2_, Prostaglandin E_2_; SCF, Stem Cell Factor; SNO cell, spindle-shaped N-cadherin+CD45- osteoblastic cell; TNF-α, Tumors Necrosis Factor α; TPO, Thrombopoietin.

The bone marrow is a heterogeneous environment that contains various hematopoietic and non-hematopoietic cells, including HSCs and MSCs, also called stromal stem cells (SSCs) (Table 4) [86]. HSCs nest in hematopoietic niches of the BM, but their proliferation and quiescence are under the control of non-hematopoietic niches. However, under stress, they can migrate to different organs like the spleen to continue hematopoiesis [87]. The hematopoietic niche is divided into the endosteal niche and vascular niche (Figure 1) [88]. These two HSC niches differ in many aspects, including calcium levels, oxygen pressure, pH, and cellular variability [88]. Endosteal niches contain quiescent and radiation-resistant HSCs [88], whereas both quiescent and proliferating HSCs can be found within the vascular niche [88]. HSC niches are regulated by non-hematopoietic cells to produce a wide variety of blood cells [87], and MSCs form a primary part of the non-hematopoietic BM niche [89]. These cells are responsible for regulating various functions of HSCs, such as proliferation, differentiation, adhesion, and quiescence through deploying different cytokines, chemokines, and adhesion molecules [89].

In the normal BM microenvironment, HSCs are mostly in a quiescent phase (G0) through the action of factors like stem cell factor (SCF), transforming growth factor β (TGF-β), platelet factor 4 (PF4, CXCL4), angiopoietin-1 (ANGPT1), and thrombopoietin (TPO), and this quiescence is considered a protective mechanism against the destructive effects of the environment and chemotherapy [90]. In addition, SDF-1 (CXCL12) and its receptor CXCR4, both important for HSC nesting, are incorporated with the MSC-secreted cytokines, interleukin (IL)-6 and IL-8, to promote HSC survival [91,92]. Other complementary factors in HSC nesting include VCAM-1, extracellular matrix (ECM), selectins, and hyaluronic acid [91,92]. Finally, NOTCH ligand (NOTCH-L), IL-7, erythropoietin (EPO), and other factors direct the fate and terminal differentiation of cells [93]. Cross-talk and interrelationship between immune cells, dendritic cells (DCs), HSCs, and myeloid-derived suppressor cells (MDSCs) within the bone marrow niche make a regulatory network for apoptosis, proliferation, HSC protection, and homeostasis [61,94]. This cooperation between myeloid and lymphoid lineages regulates HSC differentiation, self-renewal, and proliferation to inhibit leukemia development [61].

**Table 4:** The function of various cellular components of the BM in normal and AML status.

Cell	Normal Function and Products	Role in AML	Refs
Adipocyte	1. Increases in adulthood 2. Adipokine and Adiponectin 3. Hematopoiesis negative regulation	1. Leukemic cells proliferation 2. Increased adipokinase during leukemia 3. Leukemic cell pro-survival	[44,62,87,89,95]
Endothelial cell	1. Notch L 2. E-selectin, P-selectin 3. Vascular cell adhesion molecule 1 (VCAM 1) 4. Intercellular adhesion molecule 1 (ICAM -1)	1. Vascular endothelial growth factor (VEGF) production and Granulocyte-macrophage colony-stimulating factor (GM-CSF) (potential mitogen) stimulation 2. AML progression	[87,89,95,96]
Osteoblast	1. N-Cadherin 2. Osteopoietin 3. SCF 4. CXCL12 5. HSC niche establishment	1. Osteogenesis augmentation 2. AML initiation and progression	[44,86,89,97]
CXCL12-abundant reticular cells (CAR cells)	1. Stromal cell-derived factor 1(SDF-1) 2. VCAM-1 3. E-/P-Selectin 4. CD44 5. Platelet-derived growth factors (PDFG)	Pro-survival	[44,62,87,96]
Regulatory T cells (T-reg)	1. IL-10 2. IL-35 3. Inhibits immune reactions against stem cells	1. Up-regulated in AML patients 2. AML leukemic cells induce IL-10 secreting T regulatory (iTreg) cells and natural T regulatory (N-Treg) cells through inducible co-stimulator ligand (ICOSL) expression.	
Fibroblast	1. Cancer-associated fibroblasts (CAFs) 2. Growth differentiation factor 15 (GDF15) 3. IL-8	Chemotherapy resistance	[44,95,98]

## 6. Role of the BM Microenvironment in AML and Therapy Resistance

Leukemic cells charter a highly disciplined and complex network within the BM microenvironment, especially MSCs, in order to survive and thrive. The BM microenvironment provides leukemic cells with sites to adhere to and tools for suppression of the immune system. Some studies have demonstrated that different aspects of leukemic cell characteristics, such as survival, invasion, growth, angiogenesis, proliferation, apoptosis, and signaling pathways are directly affected by non-hematopoietic cells [52,84,89,93,99,100,101,102,103]. Various cellular components, cytokines, and chemokines that impact AML initiation and therapy resistance at the cellular and molecular level are shown in Table 4 and Table 5.

AML alters the function of the BM stromal cell (BMSC) population to reshape the BM microenvironment, which in return promotes AML tumor cell survival and proliferation. AML cells induce senescence in BMSCs, as demonstrated by increased p16INK4a, β-Galactosidase, and IL-6, and reduced Lamin B [137]. The p16INK4a-driven senescence in BMSC increases the survival and proliferation of AML cells in return [138]. The increased p16INK4a in BMSC seems to be independent of direct cell-cell contact, and is rather due to cytokine secretion. In vivo and in vitro data showed that depletion of non-malignant BMSCs has anti-leukemia activity, and can therefore be considered a therapeutic option [138]. Induction of p16INK4a in BMSCs and subsequent senescence has been shown to be due to superoxide, a type of reactive oxygen species (ROS). The production of ROS by AML cells appears to be through the activity of NADPH oxidase 2 (NOX2) [138].

During leukemic transformation within the BM niche, MSCs are altered to make the entire niche appropriate for leukemogenesis [52]. The close relationship between leukemic cells and the stromal cells of the BM is essential for the development of drug resistance [88]. Stromal cells utilize two mechanisms to induce drug resistance, including soluble factor-mediated drug resistance (SM-DR) and cell adhesion-mediated drug resistance (CAM-DR) [139]. SM-DR includes soluble factors like CXCL12, vascular endothelial growth factor (VEGF), IL-6, fibroblast growth factor (FGF), granulocyte-colony stimulating factor (G-CSF), and other factors mentioned in Table 6. CAM-DR, on the other hand, is caused by direct cell-cell interactions (Table 6) [139]. In vitro assays demonstrated that the co-culture of AML and stromal cells leads to stroma-derived soluble factor (SDSF) secretion, resulting in MAPK/ERK kinase (MEK) pathway activation in leukemic cells and consequently increased survival [104,140]. Additionally, co-culture of apoptosis repressor with caspase recruitment domain (ARC)/IL-1β-expressing MSCs with AML cells upregulates cyclooxygenase-2 (COX-2) and prostaglandin E_2_ (PGE_2_) expression in MSCs. The IL-1β-mediated induction of PGE_2_ secretion from MSCs leads to β-catenin activation and the induction of malignant transformation of HSCs, up-regulation of ARC, and enhanced chemotherapy resistance in AML [141]. Conversely, β-catenin blockage leads to ARC decline and chemo-sensitization [141].

One of the findings in the BM of AML patients is the failure of normal hematopoiesis. BM failure is not due to depletion of HSC numbers, but rather due to failure of the BM to produce sufficient numbers of progenitor cells [152]. The MSCs seem to play a major role in blocking the transition from HSCs to progenitors in the BM of AML patients. Recent data suggest that hypoxia in the BM of AML patients activates hypoxia-associated molecules, such as stanniocalcin1 (STC1), which is secreted from MSCs and increases the stemness of normal HSCs, thereby preventing differentiation [153].

**Figure 2:**
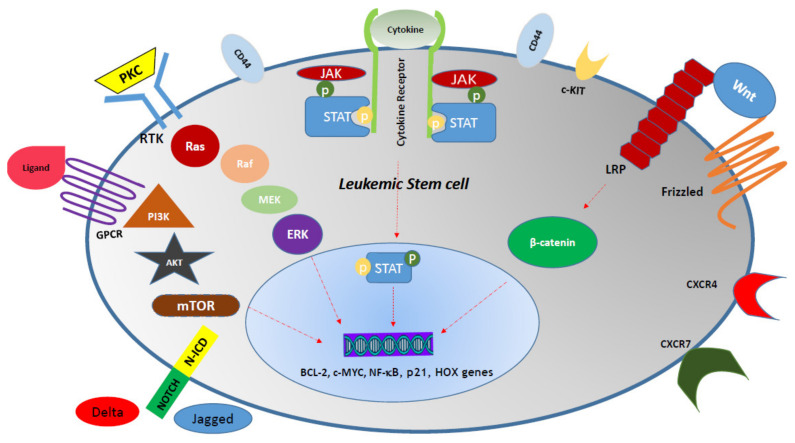
Activation of different signaling pathways in a leukemic stem cell. AKT, PKB (Protein kinase B); BCL2, B-cell lymphoma 2; GPCR, G-protein-coupled receptor; JAK, Janus kinase; LRP, lipoprotein receptor-related protein; mTOR, mechanistic target of rapamycin; N-ICD, Notch-intracellular domain; NF-κB, Nuclear factor-kappaB; PI3K, Phosphoinositide 3-kinases; PKC, Protein kinase C; RTK, Receptor tyrosine kinases; STAT, Signal transducer and activator of transcription.

Signaling pathways are another part of this regulatory network, allowing the microenvironment to control leukemia cell behavior and vice versa. Interruptions in any of these pathways may lead to cross-talk imbalance and the development of leukemia [154,155,156]. Dysregulation of various signaling pathways have been shown to be responsible for the aberrant self-renewal in leukemic cells, leading to poor prognosis and chemotherapy resistance in many AML cases [157,158,159]. Some effects of signal pathway disruption are presented in Table 6 and Figure 2.

A recent report by Forte et al. showed the role of nestin-positive (nestin^+^) MSCs in AML development and resistance to chemotherapy [160], providing a rich niche for the HSCs and LSCs. In contrast with chronic myeloid leukemia (CML), where there is a reduced number of nestin^+^ MSCs [161], there is an enrichment of nestin^+^ cells in AML bone marrow, and this enrichment is essential for the viability and proliferation of AML cells in vitro and in vivo [160,162]. In addition to their role in the development of AML, nestin^+^ MSCs were demonstrated to induce resistance to chemotherapy through enhanced glutathione (GSH)-peroxidase (Gpx) activity. AML LSCs were recently shown to increase their metabolic activity through enhanced oxidative phosphorylation (OXPHO) and increased tricarboxylic acid (TCA) cycle in mitochondria. This increased reliance on mitochondrial activity is further provided by transfer of mitochondria from nestin^+^ MSCs directly to the AML cells. Increased metabolism leads to increased ROS production, which must be controlled or it is lethal to the cells, and therefore the antioxidant glutathione pathway is induced in AML cells by nestin^+^ MSCs through activating GSH-Gpx [160].

Indirect connections between leukemic cells and the microenvironment is in part regulated by cellular vesicles which are divided into exosomes, exomers, microvesicles, and apoptotic bodies, based on their size or source [163,164]. Exosomes are secreted by normal and/or leukemic cells, and in contrast to their size (30–100 nm), contain various mRNAs, microRNAs, long non-coding RNAs, and proteins (i.e., cytokines) that play important roles in regulating cell proliferation, differentiation, and apoptosis [165,166]. Exosomes carry factors like Fas Ligand (FAS-L), NPM1, FLT3, Matrix Metallopeptidase 9 (MMP9), insulin-like growth factor type 1 receptor (IGF1-R), CXCR4, and chaperones to alter the BM microenvironment, improve leukemic cell survival, and extrinsically mediate drug resistance in primarily sensitive AML [165,167,168]. The exosomes are identified by markers such as ALG-2 interacting protein X (ALIX), CD63, CD81, CD9, syndecan-1, tumor susceptibility gene 101 (TSG 101), major histocompatibility complex (MHC) molecules, and heat shock protein 70 (HSP 70) [165].

Recent data suggests that other tissue microenvironments may also contribute to drug resistance in AML. For instance, it was reported that the liver niche promotes proliferation of resident leukemic cells and prevents their apoptosis through regulating their polyunsaturated fatty acid (PUFA) metabolism, leading to activation of the ERK pathway to promote the stability of the anti-apoptotic proteins, BCL-2 and BCL-XL. Additionally, infiltrating AML cells caused damage to hepatocytes, resulting in the secretion of cytidine deaminase (CAD) from the damaged hepatic cells. The released CAD destroys chemotherapeutic agents, thereby leading to drug resistance. [169].

## 7. Metabolic Pathways, AML LSC Survival, and Resistance to Therapy

Venetoclax in combination with hypomethylating agents has been approved for the treatment of both newly diagnosed and relapsed/refractory AML patients [170]; however, 30% of patients show primary resistance and many others develop resistance following treatment [171]. Primary AML cells cannot effectively use common metabolic fuels such as glucose or fatty acids, but have an aberrant reliance on the uptake and catabolism of amino acids to drive the TCA cycle and promote OXPHOS. The combination of Venetoclax and Azacytidine (ven/az) inhibits amino acid metabolism, leading to reduced OXPHOS and LSC death [172]. However, ven/az is ineffective at relapse because the LSCs change their metabolic preferences and requirement for amino acids. At relapse, LSCs increase their energy production and, in addition to amino acids, use fatty acids as sources for the increased activity of the TCA cycle. The enhancement of TCA cycle activity depends on nicotinamide adenine dinucleotide (NAD^+^)-dependent TCA cycle enzymes, which require higher NAD^+^ levels for their activity. NAD^+^ is produced through salvage pathways from nicotinamide during relapse [173]. Primary AML patient cells were found to produce high levels of superoxide, a phenomenon that could be related to cell proliferation [174]. AML LSCs and their progeny have been shown to have a greater mitochondrial mass and higher rates of oxygen consumption compared with normal HSCs. There are increasing amounts of data in the literature showing a significant role for mitochondria in both AML pathogenesis and resistance to therapy. Mitochondria contain complexes that regulate protein levels by eliminating excess or damaged proteins. One of the 15 identified proteases for eliminating damaged proteins in the mitochondria is caseinolytic protease P (ClpP) [175]. ClpP maintains the integrity of OXPHOS, and its inhibition results in an increase of misfolded proteins in the respiratory chain, leading to respiratory dysfunction in AML cells [176]. However, hyperactivation of ClpP can also be toxic to cells. The activation of ClpP by ONC201 and ONC212 was shown to induce apoptosis in primary AML cells with little effect on normal HSCs [177]. Primary AML patients with higher ClpP expression were shown to be more sensitive to ClpP activators compared with samples that have lower-than-average expression levels. Activation of ClpP selectively degrades the respiratory chain similarly in normal HSCs; however, the greater sensitivity of AML cells reflects the enhanced reliance of AML cells on OXPHOS and lower spare reserve capacity in their respiratory chain [177].

Targeting different components of the mitochondria has been suggested as a strategy to overcome resistance in patients treated with ven/az. The caseinolytic peptidase B protein homolog (CLPB) protein, a mitochondrial AAA+ ATPase chaperone, was one of the genes shown to be upregulated in primary AML, and was further upregulated upon acquisition of Venetoclax resistance [178]. Cheng et al. showed that CLPB maintains the mitochondrial cristae structure through its interaction with the cristae-shaping protein, OPA1, and if lost, promotes apoptosis by inducing cristae remodeling and mitochondrial stress responses. This finding suggests that targeting mitochondrial architecture may provide a promising approach to circumvent Venetoclax resistance [178].

In a study by Hole et al., 65% of AML patients showed significantly elevated superoxide production compared with normal controls, which was shown to occur through the function of NOX family members [55]. The enhanced ROS formation promotes cell proliferation and migration and thereby contributes to leukemic cell transformation [179,180]. In normal cells, ROS-induced stress results in activation of stress-activated protein kinase (SPARK). p38^MAPK^ is a SPARK that is activated by ROS, resulting in cell cycle arrest. The high level of ROS in primary AML blasts is associated with defective p38^MAPK^ stress signaling [174]. This means that, in spite of high ROS production, the AML blast cells do not undergo cell cycle arrest. The elevated ROS levels have not been shown to be limited to particular AML subtypes [174]. Among the NOX family, mainly *NOX2* expression in primary AML blasts has been shown to be correlated with superoxide production [174]. The generated superoxide by NOX is converted to H_2_O_2_ by superoxide dismutase. Primary AML cells constitutively generate H_2_O_2_, which promotes the proliferation of both AML blasts and cell lines [174], and therefore NOX2 may be essential for the viability and proliferation of AML cells [181]. However, a different mechanism for oncogenicity of NOX2 in AML was reported by Adane et al., who demonstrated that the NOX2 complex is strongly expressed in LSCs and its expression is important for LSC self-renewal [182]. The role of NOX2 at inducing self-renewal was shown to be through activation of FOXC1. Inhibition of NOX2 in the LSCs of an AML mouse model reduced the dynamic of mitochondrial and glycolytic metabolism, indicating that suppression of NOX2 could reduce the core metabolic pathways in AML cells and be a therapeutic option for eradicating AML LSCs [182].

## 8. Concluding Thoughts

AML is a heterogeneous disease that has a poor prognosis, especially in older individuals. Both intrinsic and extrinsic factors of leukemic cells and signals from the BM microenvironment play a role in disease pathogenesis and response to therapy. In recent years, many different enzymes, transcription factors, signaling pathways, and components of the microenvironment have been shown to contribute to LSC survival and drug resistance in AML, and thereby represent novel targets for therapy. As a result, several different targeted therapies have been developed for the treatment of AML. Although these types of medications improve the outcome of many AML patients, some still have an unfavorable response, meaning that we have much more to discover in order to cure this incredibly challenging disease. In the future, personalized medicine will be required to eradicate this disease, in which a patient is treated based on their individual mutation status and drug sensitivity. Eradication of AML will rely on the realization of new target inhibitors and the use of multiple drugs in personalized medicine approaches. Finally, the heterogeneity of the disease highlights the importance of personalized medicine and the need for new diagnostic methods.

## Figures and Tables

**Table 1:** WHO classification of AML subtypes [27].

Number	Genomic Classification of AML	Rate
1	NPM1-mutated AML	27%
2	AML with mutated chromatin and/or RNA-splicing genes which include (RUNX1, MLL, SRSF2, ASXL1, STAG2)	18%
3	AML with TP53 mutations and/or chromosomal aneuploidy	13%
4	AML with inv (16) (p13.1q22) or t(16;16) (p13.1; q22); CBFB–MYH11	5%
5	AML with biallelic CEBPA mutations	4%
6	AML with t (15;17) (q22; q12); PML–RARA	4%
7	AML with t (8;21) (q22; q22); RUNX1–RUNX1T1	4%
8	AML with MLL fusion genes; t(x;11) (x; q23)	3%
9	AML with inv (3) (q21q26.2) or t (3;3) (q21; q26.2); GATA2, MECOM (EVI1)	1%
10	AML with IDH2R172 mutations and no other class-defining lesions	1%
11	AML with t (6;9) (p23; q34); DEK–NUP214	1%

**Table 2:** Medications with the purpose of AML targeted therapy.

Function	Name	Target	Mechanism	FDA Approved	Refs
IDH1 inhibitor	Ivosidenib	IDH1	Myeloblast differentiation induction through isocitrate dehydrogenase 1 (IDH1) inhibition and 2-hydroxyglutarate (2-HG) blockage	Yes	[46]
IDH2 inhibitor	Enasidenib	IDH2	Myeloblast differentiation induction through isocitrate dehydrogenase 2 (IDH2) inhibition and 2-HG blockage	Yes	[46]
FLT3 inhibitor	Gilteritinib	FLT3-TKD	FLT3-I inhibition AXL receptor tyrosine kinase inhibition FLT3-TKD and FLT3-D835 TKD receptor antagonist	Yes	[47]
	Quizartinib	FLT3-ITD	FLT3 second generation inhibitor Tumor cell apoptosis inducer	No	[47,48]
Antibody drug conjugate (ABDC)	Gemtuzumab ozogamicin (GO)	CD33	Anti-CD33 monoclonal antibody conjugated with cytotoxin	Yes	[46]
Selective E-selectin antagonist	Uproleselan (GMI-1271)	E-selectin	Chemotherapy sensitizer	No	[46]

**Table 3:** Transcription factor roles in AML.

TF	Effects	Therapeutics	Refs
NF-E2 related factor-2 (NRF2)	1. Reactive oxygen species (ROS) neutralization 2. Chemotherapy resistant 3. Antioxidant response element (ARE) up-regulation	Brusatol	[70,71]
CCAAT/enhancer binding protein alpha (C/EBPα)	1. Tumor suppressor 2. Activated by TP53-KLF4 3. Down-regulated in AML due to TP53 down-regulation 4. Drug resistance 5. CSF3R, MPO, and ELANE up-regulation	ICCB280 NSC23766 OICR-9429 C/EBPA-siRNA	[71,72]
TP53	1. Tumor suppressor 2. Down-regulated in AML 3. Severe drug resistance 4. BAX and CDKN1A up-regulation	PRIMA-1 PRIMA-1MET SAR405838 AM-8553 AMG232 MK-8242 DS-3032b CGM097	[71,73]
c-MYC	Up-regulated in AML 1. Leukemic cells proliferation enhancement 2. Chemotherapy resistance 3. BCL-2, CDKN1A and CCND1 up-regulation	IIA6B17 NY2267 MYRA-A 10074-G5 Mycro3 JQ-1	[71,74]
STAT3	Up-regulated in AML 1. Chemotherapy resistance 2. Pro-survival 3. Proliferation enhancement 4. Anti-apoptotic 5. BCL-2, BCL-XL, Mc1-1, cyclin D1, and c-MYC up-regulation	Galiellalactone	[71,75,76]
Krüppel-like factor 4 (KLF4)	1. Tumor suppressor 2. Cell cycle arrest by CDKN1A suppression 3. Down-regulated in AML (NPM1-mutant) 4. Down-regulation is correlated with chemoresistance 5. P21, P27 up-regulation 6. Suppressed by metal-regulatory transcription factor 1 (MTF-1)	APTO-253	[69,71,72,77]
cAMP response element-binding protein (CREB)	Up-regulated in AML 1. Pro-survival 2. Anti-apoptotic 3. Chemotherapy resistance 4. Up-regulates BCL-2 5. Up regulates transcription of numerous gens such as c-fos, junB, and egr-1	STF-017794 STF-038533 STF-046536 STF-046728 STF-055910	[69,71,78,79,80]
PU.1	Up-regulated in AML 1. Up-regulates CSF1R, IL7R, CD11b, M-CSFR, GM-CSFR, G-CSFR 2. Hematopoiesis defect in AML	DB2313 DB2115 DB1976	[71,81]
Runt-related transcription factor 1 (RUNX1)	Up-regulated in AML 1. Up-regulates C/EBPα, PU.1, and cell cycle progression 2. Down-regulates TP53	Chb-M Chb-50	[71,82]
NF-κB	Up-regulated in AML Poor prognostic factor 1. Up-regulates BCL-2 and BCL-XL 2. Pro-survival 3. Feed-back positive effect with TNF-α in AML	Bortezomib (FDA)	[83,84,85]

**Table 5:** BM cytokine and chemokine network interrelationship in AML.

Receptor	Cell(s)	Ligand	Ligand Source	Normal Function	Expression in AML	Refs
CXCR4	1. Most immune cells 2. AML leukemic cells	SDF-1 (CXCL12)	1. MSC 2. Leukemic cells	1.Chemotaxis 2. Migration 3. Pro-survival	1. Chemotherapy resistance 2. Pro-survivalthrough PI3K/AKT and MEK/ERK activation	[44,95,102,103,104]
VCAM-1 (CD106, fibronectin)	Stromal cells	Very late antigen 4 (VLA-4)	1. HSC and hematopoietic progenitors 2. Monocytes (MO) 3. Leukemic cells 4. Myeloid cells 5. Immature dendritic cells 6. Neutrophils 7. Eosinophils 8. Immature mast cells 9. Endothelial cells	1. Adhesion 2. Pro-survival 3. Proliferation	1. Pro-survival 2. Proliferative 3. NF-κB activation 4. Chemotherapy resistance 5. MRD and relapse	[62,95,105,106]
RANK	NK cell	RANKL or Tumor necrosis factor-receptor (TNF-R)	1. Stromal cells 2. Osteoblast 3. Activated lymphocyte 4. Leukemic cells	Bone remodeling	NK cell inhibitory	[44]
c-MPL (CD 110)	1. HSC 2. Megakaryocyte (MK) 3. Chronic myeloid leukemia (CML) 4. AML leukemic cells	TPO	1. Liver 2. Kidney	1. HSC quiescence 2. Thrombopoiesis	Chemotherapy resistance	[87,107]
Vascular endothelial growth factor receptor(VEGFR)	1. MO 2. MQ 3. Vascularendothelial cells (VEC) 4. Lymphoid endothelial cells (LEC) 5. HSC	1. VEGF 2. PIGF	1. Stromal cell 2. MK 3. HSC 4. Leukemic cells	1. GM-CSF stimulation 2. Angiogenesis 3. Metabolichomeostasis 4. Proliferation 5. Migration 6. Tubulogenesis	1. Anti-apoptotic 2. Chemotherapy resistance	[32,95,108]
E-Selectin	1. Endothelial cells 2. Stromal cell	CD44	1. HSC and Hematopoietic progenitors 2. T cells 3. Leukemic stem cells 4. Stromal cells	1. HSC pro-survival 2. Proliferation of HSCs	1. E-selectin: chemotherapy resistance 2. CD44: Pro-survival	[95,104,105,109]
IL-1R1	1. Most hematopoietic and non-hematopoietic cells 2. AML leukemic cells	IL-1β	1. Myeloid lineage 2. Leukemic cells 3. EC 4. MSC 5. MQ	1. Pro-inflammatory 2. Hematopoiesis regulation	1. Pro-survival 2. Pro-proliferative 3. Sometimes feedback positive 4. Association with endogenous IL-1β related to apoptosis resistance	[110,111,112,113,114,115,116]
TNFαRI (p55 or p60)	A broad spectrum of different cell types like AML cells	TNF-α	1. CD8/ CD4 T cell 2. NKT cells 3. Neutrophils 4. Macrophage 1 (MQ_1)_ 5. LSCs 6. MSCs	Pro-inflammatory	1. Pro-survival 2. Chemotherapy resistance 3. NF-κB activation	[44,110,113,117,118,119,120]
IFNGR1,2	1. Widelydistributed on various cell types 2. LSCs	IFN-ϒ	Most immune cells	Pro-inflammatory	1. Anti-leukemic 2. Anti-proliferative 3. Antigen presentation through MHC I/II augment 4. Nitric oxide (NO) and reactive oxygen species (ROS) mediators, NADPH, and inducible nitric oxide synthase (INOS) production	[110,118,121,122,123]
IL-10R	1. AML leukemic cells 2. T cells 3. B cells 4. NK cells 5. Epithelial cells 6. Endothelial cells 7. Plasmacytoid DCs 8. Peripheral blood mononuclear cells (PBMCs)	IL-10	1. T helper 2 (TH 2) 2. BM-MSCs 3. Macrophage 2 (MQ_2_) 4. T-reg 5. B cells 6. MO 7. Thymocytes	Anti-inflammatory TH1 suppressor	1. Growth arrest-specific gene 6 (Gas6) up-regulation 2. Pro-survival 3. Chemotherapy resistance	[118,123,124,125,126,127,128,129]
TGF-βR	1. T cell 2. Hematopoietic progenitor cells 3. AML leukemic cells	TGF-β	1. T-reg 2. MQ2 3. MSC 4. Endothelial cells 5. Platelets 6. PBMCs	1. Anti-inflammatory 2. Proliferation 3. Migration 4. Pro-survival 5. Growth and differentiation inhibition of hematopoietic progenitor cells	1. Anti-proliferative 2. IL-1β, IL-6, GM-CSF, and granulocyte colony-stimulating factor (G-CSF) production 3. Reduction in AML	[110,118,126,130]
IL1R1	1. Most hematopoietic and non-hematopoietic cells 2. AML leukemic cells	IL-1Ra	1. MQ 2 2. MO 4. Neu 6. Fibroblasts 7. Chondrocytes	1. Anti-inflammatory 2. IL-1 antagonist	Leukemic cell colonization inhibitor	[110,112,131,132]
IL-35R	1. Effector T cells 2. CD4^+^ T-reg 3. AML leukemic cells	IL-35	1. T-reg 2. DCs 3. B-reg 4. sometimes in endothelial cells, monocytes and smooth muscle cells	1. Anti-inflammatory 2. Inhibits T cell proliferation 3. Transformation of T cells to iTreg	1. Anti-apoptotic 2. Proliferation 3. Weak prognosis 4. AML progression	[110,118,133]
PD1 (CD279)	Lymphocytes	Programmed death-ligand 1 (PDL1) (CD274) (B7-H1)	1. T-reg 2. Follicular T cells (FTC) 3.MQ 4. Dendritic cell (DC) 5. placental syncytiotrophoblasts 6. MO 7. AML leukemic cells	T cell activation and proliferation inhibitor	1. Pro-survival 2. Weak prognosis	[118,134]
Lymphocyte activation gene-3 (LAG3)	T cell	MHC II	APCs	T cells inhibitory	1. Correlation with programmed death-1 (PD1) 2. Increased activity of leukemic cells	[118,135]
Galectin-9 (Gal-9)	1. AML LSC 2. Lymphocytes 3. Spleen 4. Thymus	T-cell immunoglobin mucin-3 (TIM-3)	1. AML leukemic cells 2. MO 3. DC 4. Some of T cells 5. NK cells 6. Myeloid pre-leukemic progenitors Not in normal HSCs	1. TH1 inhibitory 2. DC maturation 3. TNF-α secretion from monocytes 4. Innate immune system activation	Strong self-renewal signaling through TIM-3/Gal-9 autocrine loop, NF-κβ and β-catenin signaling Up-regulated in pre-leukemic disorders	[136]
Cytotoxic T-lymphocyte antigen-4 (CTLA-4) or (CD152)	1. T cells 2. AML leukemic cells	Β7-1 Β7-2	Antigen-presenting cells (APCs)	T-cell inhibitory and tolerance induction	1. AML relapse and MRD 2. Immune evasion Blockage leads to sensitivity to cytotoxic T lymphocytes (CTL)	[134,137]

**Table 6:** Signaling pathways related to AML drug resistance.

Signaling Pathway	Leukemic Effect	Mechanism	Therapeutics	Activator Ligand (L) Receptor (R)	Mediators (M) Target (T)	Refs
JAK/STAT	Chemo-therapy resistance	1. Proliferation 2. Pro-survival	1. Ruxolitinib (FDA) 2. Ruxolitinib 3. Pacritinib 4. Lestaurtinib 5. Fedratinib 6. Momelotinib	L: TPO/MPL/G-CSF R: Cytokine receptor superfamily	M: JAK2, STAT3, STAT5, TYK2 T: p21, Mcl-1, PIM1, BCL-2, BCL-XL	[142,143]
Notch1	1. Poor prognosis 2. Chemotherapy resistance	1. Rb phosphorylation 2. C-MYC and BCL-2 up-regulation 3. Pro-survival 4. Proliferation 5. Connection to Delta-1 leads to NF-κB pathway activation	GSIs (GSI-IX and GSI-XII)	L: Deltalike1,4 Jagged1 R: NOTCH1	M: Notch intracellular domain of Notch (N-ICN) T: 1. CSL activity Hes family: HES1, HES5 Hes-related repressor proteins (Herps) family: HERP2 2. DELTEX1	[144,145]
Hedgehog (Hh)	1. Poor prognosis 2. Chemotherapy resistance	Activated in AML through GLI1 and SMO up-regulation	1. LDE225 (Sonidegib) 2. PF-04449913 (Glasdegib) 3. Vismodegib (GDC-0449) 4. BMS-833923 (XL139) 5. GANT-61	L: Hh proteins R: PTCH1 and SMO	M: GLI1 T: BCL-2, SNAIL, RAS, TGF-β, c-MYC	[146]
Ras/Raf/MEK/ERK	1. Chemotherapy resistance 2. Leukemic cell survival	1. Anti-apoptotic 2. Pro-survival through Raf-1 downstream molecule phosphorylation	1. L-779,450 2. ZM 336372 3. Bay 43-9006 4. Geldanamycin 5. Coumermycin 5. Dasatinib 6. PD98059 7. U0126 8. PD184352 9. ARRY142886	L: 1. Ras proteins (Ha-Ras, N-Ras, Ki-Ras 4A, Ki-Ras 4B) 2. Protein kinase C (PKC) R: Receptor tyrosine kinases (RTK)	M: Raf-1, A-Raf and B-Raf T: 1. Transcription factors, including Ets-1, c-Jun and c-MYC CREB NF-κB 2. Bad, Bim, Mcl-1, caspase 9, BCL-2	[147,148]
Phosphatidy-linositol 3-kinase (PI3K)/Akt/mTOR	1. Poor prognosis 2. Chemotherapy resistance	1. Glycolysis up-regulation 2. Proliferation 3. Pro-survival	1. Ridaforolimus 2. Sirolimus (Rapamycin) 3. Everolimus 4. Temsirolimus	L: Wide variety of extracellular stimuli R: G-protein-coupled receptors (GPCRs) RTK, various integrins, B and T cell receptors	M: Akt, mTOR T: p70S6K, S6RP, 4EBP1	[53,149]
Wnt	1. Poor prognosis 2. Chemotherapy resistance	1. LSC self- renewal 2. AML progression	1. Celecoxib 2. CWP232291 3. LY2090314 4. PRI-724 5. Sulindac	L: Wnt1 Wnt3a, PCP R: Frizzled (FZD) and lipoprotein receptor-related protein (LRP)	M: β-catenin, Ca2^+^ T: cyclin D1, c-MYC, Hox genes, MLL/ENL	[150,151]
